# Buffet hypothesis for microbial nutrition at the rhizosphere

**DOI:** 10.3389/fpls.2013.00188

**Published:** 2013-06-14

**Authors:** Martha G. López-Guerrero, Ernesto Ormeño-Orrillo, Mónica Rosenblueth, Julio Martinez-Romero, Esperanza Martïnez-Romero

**Affiliations:** Centro de Ciencias Genómicas, Universidad Nacional Autónoma de MéxicoCuernavaca, Mexico

**Keywords:** rhizosphere, speciation, root exudates, soil microbiology, bacterial genomes

## Abstract

An emphasis is made on the diversity of nutrients that rhizosphere bacteria may encounter derived from roots, soil, decaying organic matter, seeds, or the microbial community. This nutrient diversity may be considered analogous to a buffet and is contrasting to the hypothesis of oligotrophy at the rhizosphere. Different rhizosphere bacteria may have preferences for some substrates and this would allow a complex community to be established at the rhizosphere. To profit from diverse nutrients, root-associated bacteria should have large degrading capabilities and many transporters (seemingly inducible) that may be encoded in a significant proportion of the large genomes that root-associated bacteria have. Rhizosphere microbes may have a tendency to evolve toward generalists. We propose that many genes with unknown function may encode enzymes that participate in degrading diverse rhizosphere substrates. Knowledge of bacterial genes required for nutrition at the rhizosphere will help to make better use of bacteria as plant-growth promoters in agriculture.

## INTRODUCTION

Ecophysiology of root systems cannot be understood without the microbiota that colonize outside and inside roots. Bacteria and fungi may impact root physiology, produce hormones, stimulate root growth or alter its morphology. Microbes provide protection against pathogens, tolerance to abiotic stresses, resistance to insect or herbivore attack; even allelopathy may be due to root-associated microorganisms. An extensive review on the ecophysiological contributions of microorganisms to plants has been published (Friesen et al., 2011) and reviews on rhizospheric bacteria also highlight their effects on plants (van Loon et al., 1998; Bais et al., 2006; de Bruijn, 2013). Microbial endophytes (meaning residing inside the roots) may contribute to nutrient assimilation and other plant traits, however, they are normally in lower numbers than rhizospheric bacteria (Rosenblueth and Martínez-Romero, 2006; Hirsch and Mauchline, 2012) and we will focus only on the latter. Over the years, studies on root microbiota have addressed several questions such as: How are microbes selected or maintained in roots? What are the sources and resources for root microbes? How do bacteria or fungi affect root physiology? Are there key species that have a larger impact on plants? Is nutrient competition driving bacterial evolution? There are still questions without answer.

The term rhizosphere was proposed by Hiltner (1904) and refers to 1–7 mm of soil from the root surface. The rhizosphere effect is the enrichment of microbial populations at the root–soil interface. Outside roots there is a heavy colonization of bacteria (for example, 10^9^ *Rhizobium phaseoli* cells per gram of fresh maize root; Gutiérrez-Zamora and Martínez-Romero, 2001) mainly stimulated by root-derived nutrients. The microbial community itself may modify root nutrients and may contribute with resources by transforming soil material (Baelum et al., 2008), by fixing nitrogen (Fischer et al., 2012) or producing vitamins (Phillips et al., 1999; Ramírez-Puebla et al., 2013). Rhizosphere nutrients may be very variable depending on the plant (Brown et al., 2008; Haichar et al., 2008; Badri et al., 2013) and the soil biotic and abiotic conditions. There are bacterial species commonly encountered as rhizosphere colonizers but each plant species may harbor particular microbes at the rhizosphere (Lundberg et al., 2012). A complex rhizosphere community may be structured in relation to the microbial specialization for different nutrients. The diversity of nutrients available at the rhizosphere may be equated to a buffet, and distinct microbes may have preferences for some of them. Furthermore, we propose that a large proportion of products from genes highly expressed by bacteria at the rhizosphere are involved in the transport and catabolism of the various buffet entries.

## PLANT-DERIVED NUTRIENTS AT THE RHIZOSPHERE

Plants may be considered as a growth media for their microbiota (Brown et al., 2008). Root exudates determine bacterial community structure (Haichar et al., 2008) and rhizodeposits (Dennis et al., 2010) may do the same as well. Root exudates contain a large diversity of molecules (reviewed in Walker et al., 2003; Bais et al., 2006; Dennis et al., 2010; Ramírez-Puebla et al., 2013) and around 10,000 types of flavonoids are known from plants (Ferrer et al., 2008). Additionally, arabinogalactan-proteins (AGPs) that have a large proportion of carbohydrates covalently bound to polypeptides are found abundantly in exudates (Fincher et al., 1983). AGPs are considered the most structurally complex molecules in nature (Majewska-Sawka and Nothnagel, 2000).

Exudates and other plant substances may act to select microorganisms (Walker et al., 2003; Shaw et al., 2006; Badri and Vivanco, 2009; Dennis et al., 2010; Berendsen et al., 2012) as prebiotics do (Ramírez-Puebla et al., 2013); additionally, just adhesion to plant lignocellulose acts to select bacteria from the soil (Bulgarelli et al., 2012). From root extracts, the phenolic fraction was found to have an important role in conditioning bacterial communities (Badri et al., 2013). Roots have a remarkable ability to synthesize diverse secondary metabolites (Flores et al., 1999) and many complex carbon molecules (Dennis et al., 2010; Mathesius and Watt, 2011). Seeds are also a source of nutrients for plant-associated bacteria and some contain large amounts of phytate (Lott et al., 2000). Germinated seedlings provide enough sulfur in root exudates for bacterial growth (Snoeck et al., 2003).

Plants may control bacterial growth with antimicrobials such as phytoalexins (González-Pasayo and Martínez-Romero, 2000; Shaw et al., 2006), bacterial-quorum plant-produced mimics (Bauer and Robinson, 2002), or other substances yet unknown. Additionally, plant-derived substances may control bacterial metabolism (Shaw et al., 2006; Hassan and Mathesius, 2012), perhaps to the plant own benefit. On roots, bacteria exhibit a differential gene expression that varies depending on the plant (Ramachandran et al., 2011; López-Guerrero et al., 2012). The analysis of known bacterial genes expressed in the root or rhizosphere may help us deduce conditions therein. Based on the large numbers of transporters expressed by rhizospheric bacteria (Ramachandran et al., 2011; López-Guerrero et al., 2012), we propose that each bacterial species can use a wide range of the nutrients that plants provide from roots.

Root-derived nutrients may be modified by the associated microbiota directly by transforming them to new substances (Shaw et al., 2006) or by inducing changes in plant production of exudates from the interaction with the plant. Symbiosis with microbes and fungi can alter the composition of exudates (Bais et al., 2006; Scheffknecht et al., 2006).

## SOIL-DERIVED NUTRIENTS

Besides root-derived nutrients, microbes at the rhizosphere may profit from soil-derived substrates. Many soils are substrate rich especially those having high content of organic matter, not even considering man-derived soil contaminants. Soil has perhaps the highest microbial diversity of all habitats. This may be explained by soil structure, diverse soil physical characteristics, differences in pH, minerals, metals, plethora of soil microhabitats but also by an unknown large diversity of natural substances found in soil. Humic acids in soils are very complex and their diverse chemical structure has just started to be determined (Nebbioso and Piccolo, 2001). In the rhizosphere different Amadori compounds (*N*-glycosylamines) may be found that form spontaneously from decomposing plant material or by *Agrobacterium* spp. (Baek et al., 2003).

Soil is not only the depositary of plant and animal decay matter but it is also the residence of fungi, nematodes, protozoa, insects and their products, as well as human-derived recalcitrant substances, all of them constitute an enormous array of potential food for most diverse microbes. Their use would benefit not only microbes but also their plant hosts when making nutrients available. Soil bacteria have major roles in nutrient cycles. Phosphorus solubilizing rhizospheric bacteria promote plant growth (Rodríguez and Fraga, 1999) and microorganisms participate in plant mineral acquisition (Hinsinger, 1998).

## LIFE AT THE RHIZOSPHERE FROM A NUTRITIONAL PERSPECTIVE

Different rhizosphere bacteria may have preferences for distinct substrates (Shaw et al., 2006) and this would allow a complex community to be established at the rhizosphere. Different parts of the roots are colonized by different microbes and exudation and rhizodeposition varies qualitatively in different parts of the roots (Badri and Vivanco, 2009; Dennis et al., 2010). Some plants may exude more than others (Dennis et al., 2010) and maintain larger microbial populations. Results from a proteomic-based analysis suggested that bacteria may adapt to a new range of nutrients from exudates (Cordeiro et al., 2013).

We documented simultaneous assimilation of different substrates in *Rhizobium* (Romanov and Martínez-Romero, 1994; Romanov et al., 1994). This type of metabolism would be advantageous at the rhizosphere and it has been observed in rhizoremediation (González-Paredes et al., 2013). To nourish on several plant exudated substances at the same time as well as from diverse soil substances could be a characteristic of successful rhizospheric bacteria. Genes encoding enzymes for the utilization of some Amadori compounds that may be found in the rhizosphere are patchily distributed in rhizobia (Baek et al., 2005) indicating that not all bacteria have the same degrading capacities. We have compared rhizospheric bacteria to gut bacteria in the process of digesting and converting food to host usable products (Ramírez-Puebla et al., 2013).

*Pseudomonas*, *Burkholderia*, *Streptomyces*, and rhizobia have high degrading capabilities (Kontchou and Blondeau, 1992; Juhasz et al., 1996, 2003). All may be found associated to roots and their high degrading capacities may be advantageous in rhizospheres. They have also characteristic large genomes (for examples, Bentley et al., 2002; Kaneko et al., 2002; Paulsen et al., 2005; Yan et al., 2008; Ormeño-Orrillo et al., 2012) that may be in relation to their high degrading capabilities. We suggested that many rhizobial genes of unknown function participate in the catabolism of root, rhizospheric, and soil substances (Ormeño-Orrillo and Martínez-Romero, 2013) and this could apply to other soil and rhizospheric bacteria as well.

Interestingly mutants in single genes involved in nutrient usage at the rhizosphere (Rosenblueth et al., 1998; Ramachandran et al., 2011) normally do not have clear phenotypes indicating that there are other substrates available that may be used by bacteria at the rhizosphere.

In modern times, rhizospheric microorganisms are exposed as well to anthropogenic contaminants (González-Paredes et al., 2013). Rhizoremediation takes advantage of the degrading capabilities of rhizospheric microorganisms. Organic matter in soil strongly influences the fate of contaminants (Li et al., 2011).

## CONCLUDING REMARKS

After considering the large diversity of potential nutrients (from rhizodeposits, root exudates, seeds, decaying organic matter, soil, and the rhizosphere community itself) for microbes at the rhizosphere we propose a hypothesis for bacterial nutrition at the rhizosphere: a buffet hypothesis where commensals choose their food from a diversity of options. This is in contrast to the proposal of oligotrophy at the rhizosphere (Ramachandran et al., 2011). Copiotrophic rhizobia are very successful rhizosphere colonizers (Gutiérrez-Zamora and Martínez-Romero, 2001). Microbial respiration is not carbon limited in the rhizosphere (Cheng et al., 1996). Rhizosphere is a complex environment with substitutable resources. In experimental evolution in complex environments with substitutable resources, *Pseudomonas* lineages evolved as imperfect generalists that differentiate to assimilate a certain range of substrates but not all (Barrett et al., 2005), this seems to happen with microbes at the rhizosphere.

## Conflict of Interest Statement

The authors declare that the research was conducted in the absence of any commercial or financial relationships that could be construed as a potential conflict of interest.
